# Architecture and Implementation of a Clinical Research Data Warehouse for Prostate Cancer

**DOI:** 10.5334/egems.234

**Published:** 2018-06-01

**Authors:** Martin G. Seneviratne, Tina Seto, Douglas W. Blayney, James D. Brooks, Tina Hernandez-Boussard

**Affiliations:** 1 Department of Biomedical Informatics, Stanford University, US; 2 School of Medicine Research Information Technology, Stanford University, US; 3 Stanford Cancer Institute, Department of Medicine, Stanford University, US; 4 Department of Urology, Stanford University, US; 5 Department of Medicine, Biomedical Informatics, Stanford University, US

**Keywords:** Electronic Health Records, Quality Improvement, Data Collection

## Abstract

**Background:**

Electronic health record (EHR) based research in oncology can be limited by missing data and a lack of structured data elements. Clinical research data warehouses for specific cancer types can enable the creation of more robust research cohorts.

**Methods:**

We linked data from the Stanford University EHR with the Stanford Cancer Institute Research Database (SCIRDB) and the California Cancer Registry (CCR) to create a research data warehouse for prostate cancer. The database was supplemented with information from clinical trials, natural language processing of clinical notes and surveys on patient-reported outcomes.

**Results:**

11,898 unique prostate cancer patients were identified in the Stanford EHR, of which 3,936 were matched to the Stanford cancer registry and 6153 in the CCR. 7158 patients with EHR data and at least one of SCIRDB and CCR data were initially included in the warehouse.

**Conclusions:**

A disease-specific clinical research data warehouse combining multiple data sources can facilitate secondary data use and enhance observational research in oncology.

## Introduction

The rise of electronic health records (EHRs) has created a platform to conduct observational research using routinely-collected health data [1,2]. In oncology, EHR data have increasingly been used for quality benchmarking and monitoring conformance to treatment guidelines (e.g., *KRAS* testing in colorectal cancer) [3]. EHR data have also formed the basis of comparative effectiveness studies, evaluating the real-world impact of particular investigations and interventions in oncology care [4,5].

One consistent challenge is the incompleteness of the electronic health record, with most studies only capturing data from patient encounters within a single health system [6]. This introduces biases such as skewing towards patients who receive the bulk of their care in a tertiary setting [7]. A second major challenge is the lack of structured data in many EHR records. Estimates suggest that up to 80 percent of EHR data is in unstructured form – in the text of clinical notes, radiology and pathology reports [8]. Consequently, many critical data fields for oncology research, such cancer stage, histopathologic biomarkers, smoking and functional status, are not routinely captured as structured data. In one study of lung cancer patients, for example, determining smoking status using only structured data from International Classification of Disease (ICD-9) codes had an accuracy of only 52 percent [9].

In oncology, there has been a recent push towards establishing research data warehouses to support precision medicine by integrating standard EHR data with outcomes, imaging data, and molecular and genomic analyses. Some have been enterprise-wide databases across multiple cancer types [13,17], while others have focused on particular tumor types [18]. As the data sources and formats can vary significantly between tumor types, the challenge of consolidating information from diverse data sources is often made simpler by focusing on a particular disease entity. The Oncoshare project created a hybrid research database for breast cancer, linking data from two hospital EHRs and our California Cancer Registry [19]. There were various technical challenges such as unifying differences in the data models (semantic interoperability) and resolving conflicting data points; however the resultant dataset provides a rich source of clinical histories at a level of granularity far beyond a single registry. At a larger scale, the American Society of Clinical Oncology (ASCO) has CancerLinQ, a data-sharing platform that integrates data from EHRs across different levels of care and will incorporate Surveillance, Epidemiology, and End Results (SEER) registry data [20].

The creation of clinical research data warehouses in parallel to the EHR allows supplementation and curation of EHR data using additional data streams in order to construct more comprehensive research cohorts [10]. These additional data streams may include external data from cancer registries, other health systems, insurance claims, and patient-reported outcomes; and may include new data fields extracted from the clinical text in the local EHR using natural language processing (NLP) [11,12]. The role of a warehouse is not merely to aggregate data, but also to validate data integrity, consolidate fields appropriately and otherwise curate data in order to avoid redundancy [13,14]. Large data aggregation efforts such as the cancer Biomedical Informatics Grid (caBIG), the Informatics for Integrating Biology at the Bedside (i2b2) architecture, and the Oncology Research Information Exchange Network (ORIEN) have streamlined the process of integrating data streams and provided clinicians and researchers tools to frame a question, build a cohort, and extract relevant data [15,16,21]. Other data sharing initiatives have emerged across academia and the private sector, including the Observational Health Data Sciences and Informatics (OHDSI) consortium, ASCO’s CancerLinQ, and Flatiron Health (New York City, N.Y.). The latter two platforms require a manual curation process for local EHR data to be added to the central repository. High quality local databases can support many of these multi-site initiatives.

We outline the creation of a clinical research data warehouse for prostate cancer which links data from the EHR of a tertiary academic medical center, institutional cancer registry, and state-wide cancer registry, and has the capacity to add new data streams such as NLP-derived data and patient-reported outcomes. The aim is to enrich and curate EHR data to reduce the rate of missing data, facilitate curation, and drive observational research in oncology. This framework can be incorporated into the workflow for larger projects (CancerLinQ, OHDSI, ORIEN, etc.) to facilitate efficient and meaningful secondary use of EHR and other cancer data resources.

## Methods

### Ethics

Creation of the following research data warehouse was approved by the university’s Institutional Review Board, and the State of California Institutional Review Board.

### Primary EHR data

The current Stanford University EHR is an Epic system (Epic, Verona, Wis.) installed in 2008. Data from this EHR, as well as from legacy databases prior to Epic installation extending back to 1995, are captured in the Stanford Translational Research Integrated Database Environment (STRIDE) [22]. Chemotherapy treatment data are stored in a related system (Epic Beacon) and also transmitted to STRIDE. STRIDE contains structured data, including diagnosis and procedure codes, drug exposures and laboratory results, as well as unstructured data, including discharge summaries, progress notes, pathology and radiology reports. Structured data elements are mapped to standardized terminologies including RxNorm, SNOMED, ICD and CPT. All data are transmitted from Epic systems to STRIDE daily.

### EHR cohort extraction

The first step in constructing the prostate-specific research data warehouse was to accurately phenotype patients within the Stanford EHR—that is, reliably identify patients with prostate cancer. We conducted a rule-based search using the following criteria: male subjects, an ICD-9 or ICD-10 code of prostate cancer (ICD-9: 185, 233.4 ICD-10: C61) recorded from 2005 onwards, and age above 18 years at the time of first appearance of any of the aforementioned codes. The following data were extracted for the period 2005 forward: patient demographics, diagnosis codes, procedure codes, medication exposures, lab results, pathology reports, radiology reports, and clinical progress notes. All data include a timestamp and a related encounter identifier. We manually reviewed the cohort to remove duplicates, and trim name suffixes and prefixes. High-profile patients and those who explicitly requested not to be included in research studies were excluded from the cohort.

### Internal cancer registry (SCIRDB)

The Stanford Cancer Institute Research Database (SCIRDB) is a curated database maintained by the Stanford Cancer Center for research and reporting purposes. It includes structured data fields that may not be captured by the EHR including date of diagnosis, clinical stage, pathological stage, date of death etc. SCIRDB also includes information from radiation oncology, surgical pathology and the tissue bank. A mapping is preserved to the medical record numbers (MRNs) used in the Stanford EHR. SCIRDB was queried using the MRNs of the prostate cohort identified in the EHR. Search results were filtered to ensure patients were over 18 years at the time of diagnosis and of male gender.

### CCR matching

The California Cancer Registry (CCR) contains structured data about diagnosis, histology, cancer stage, treatment and outcomes across multiple tumor types, incorporating data from health care organizations across California. We matched patients to CCR records using the name and demographic details of the EHR cohort (first name, last name, middle name, date of birth, and social security number when available). We requested 124 data fields from CCR, including detailed staging information at time of diagnosis and at restaging intervals, as well as treatment details, for the time window 2005–2016.

### Data synthesis

Where a match was identified, CCR data was combined with Stanford EHR and registry data. The only data synthesis that took place was between the SCIRDB and the CCR—a summary table was created with demographic data and tumor descriptives, which was initially populated with SCIRBD data and missing values were drawn from CCR data where available. Where discrepancies were identified, the SCIRBD data were used as the gold standard.

### Maintenance

Data from STRIDE and the SCIRDB are ported into the data warehouse on a quarterly basis. The data are collected for all existing patients within the prostate cohort, and a new query is performed on STRIDE and the Stanford cancer registry to identify additional prostate cancer patients. Data are extracted from the CCR on an annual basis. The same query is performed using the entire cohort, in order to capture new follow-up data on existing patients. The research database is managed by a dedicated database manager who supports maintenance and cohort creation for individual research studies.

### Supplementary data

Numerous research studies in our group and collaborating entities have sought to extract structured data from clinical narratives within this prostate cancer cohort. Where appropriate, these new data have been added as discrete fields in the data warehouse (e.g., evidence of urinary incontinence at the time of radical prostatectomy derived from NLP on the progress notes). In addition, where patient surveys have been collected, these have also been incorporated into the database. Prostate-specific surveys (e.g., Expanded Prostate Cancer Index survey) are collected by individual clinicians and stored in REDCap (Research Electronic Data Capture), which we automatically link to the prostate cohort. For more general surveys, such as the PROMIS Global Tool, data are captured at the hospital/clinic level and automatically integrated into the EHR system[23]. Finally, clinical trial information can be merged with the prostate cancer cohort. This is important, for example, when testing the generalizability of a small randomized clinical trial. The database architecture is flexible enough for new tables to be easily created for these supplementary data fields.

## Results

**Figure 1:**
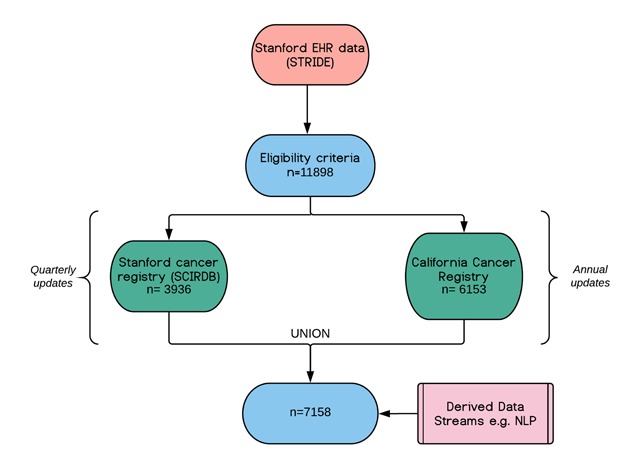
Flowchart of data sources with subject numbers at the time of database creation containing 2005–2016 data.

A total of 11,898 unique prostate cancer patients were identified in the Stanford EHR, with records spanning the following dates: 2005–2016. Of these, 3,936 were identified in the SCIRDB. This low match rate is because the registry only includes patients who had their first line of treatment at our institute. A total of 6,513 patients in the EHR-derived cohort were matched in the CCR, of which 2,217 were not included in the SCRIDB registry. Taking the union of both matched cohorts (i.e., all patients in the Stanford EHR with a record in at least one of SCRIDB or CCR) gave 7,158 patients. This was the original cohort included in the warehouse. A flowchart of the data sources is shown in Figure 1 and patient characteristics are displayed in Table 1.

**Table 1:** Patient characteristics at time of diagnosis.

*Variable*	*Value*

*N*	*7,158*
*Age in years (mean (SD))*	*65.2 (0.11)*
***Ethnicity, %***	

*White*	*69.68*
*Hispanic*	*2.15*
*Black*	*4.03*
*Asian*	*9.95*
*Other*	*14.18*
*BMI, mean (SD)*	*18.87 (0.16)*
***Stage, %***	

*1*	*10.8*
*2*	*64.7*
*3*	*11.8*
*4*	*8.6*
*Unknown*	*4.2*
*Charlson Score, mean (SD)*	*3.31 (0.03)*

## Discussion

The fragmentation of data between EHRs, registries and community care, along with the heterogeneity of how data is captured and stored, represent significant challenges in conducting clinical analytics with secondary data sources [7]. We have outlined the creation of a targeted clinical research data warehouse for prostate cancer that synthesizes data across a number of sources. This infrastructure can become an increasingly rich repository of domain specific data and derived metrics that enhances the EHR and can increase the quality and efficiency of secondary data analyses.

Most previous population-data integration efforts have combined registry data from the Surveillance, Epidemiology, and End Results (SEER) initiative with claims data from Medicare or health plans. While these combined datasets are important to study cancer epidemiology and population trends, they are often limited in scope as they do not capture the granular clinical information needed to understand complex clinical questions, for example, individual drug exposures or evidence behind clinical decisions [24]. Also, claims data often have significant lag time before their release, hindering the ability to assess emerging therapies and the early impact of guideline changes.

A significant advantage of linking EHR data with cancer registries is that this approach combines granular clinical data with important epidemiological metrics, such as tumor pathology and clinical stage. This can power comparative effectiveness research, quality benchmarking, and predictive modelling. The breast Oncoshare data warehouse, for example, has combined EHRs with gene expression data and the CCR to identify drug-class pairs associated with lower mortality [22]. By synthesizing registry and EHR data, the prostate cancer warehouse can be used to monitor adherence to guidelines and quality metrics, demonstrating the importance and utility of EHR-linked datasets.

Another advantage of aggregating EHR and registry data is in improving data completeness in both the local data marts as well as in the registries, which has been shown to be variable [25]. By incorporating the CCR data with our EHRs, we gain significantly more information on patient survival, disease progression, and treatments outside our institute. Furthermore, combining resources can help identify data discrepancies and increase data accuracy. For example, one study comparing prostate cancer data across SEER registries found that up to 18 percent of prostate-specific antigen (PSA) measures were incorrect, resulting from a misplaced decimal in the lab values [26]. Important metrics for cancer staging (e.g., digital rectal examination and Gleason score) are often poorly reported or missing from registry data [27]. In our cohort, PSA was captured in 94 percent of the EHR records, yet only 79 percent in the CCR, while Gleason score was captured in 81 percent of the EHR records, compared to 59 percent in the CCR. Warehousing efforts could be used to identify data discrepancies or missing data and supplement data marts with additional data, increasing both the accuracy and completeness of the registry and local warehouses.

A third major advantage of data aggregation is the ability to develop tools to mine the large volume of unstructured EHR data. Many of the variables captured by registries (such as clinical stage or diagnosis date) are generally not captured in structured EHR fields. Combining text notes with structured data from registries can provides the labelled training set required to develop NLP pipelines to extract these variables from clinical notes. There are also a wide range of quality metrics, such as assessment of urinary incontinence and erectile dysfunction, which are generally not captured as structured variables in either the EHR or registries. Sophisticated text-mining algorithms are being developed to extract these terms from historical data [28]. In future, this may be a way to automatically populate registries with information found in EHR unstructured data if accuracy scores (i.e., algorithm performance) are included. For example, assessment of urinary incontinence in prostate cancer patients is recognized as an important quality metric by both the American Urological Association and the National Quality Forum. We use NLP to evaluate whether urinary incontinence assessment was documented in the clinical notes and this information is stored in our database (e.g., Positive mention of urinary incontinence [Yes/No] and Accuracy Score [0–100]). While this only captures what is documented, as specified by the quality metric, such information can guide conversations on the quality of documentation and give insight into what data are being recorded, providing opportunities for data capture enhancements and clinical support technologies. These metrics could in future be fed back into registries. Such approaches for the continuous deposit of supplementary data from EHRs to cancer registries can help to power secondary research at a state or national level.

Although this resource has fueled interdisciplinary research across multiple disciplines (i.e., cancer research, biomedical informatics, computer science, etc.) there are a number of limitations to note in this data warehouse. Despite data linkage, missing or out-of-date data remains a challenge. For example, we found that many of the patients in our cohort had prostate cancer listed as an active diagnosis after completing definitive treatment and continuing disease-free for years. Further work is warranted to create an infrastructure where data quality is continually evaluated and, where possible, data fields are updated based on other data sources or manual chart review. The other key limitation is that this warehouse only encompasses data from a single academic hospital, and does not represent a scalable model for large-scale data analyses across institutions. When compared to national data repositories, such as CancerLinQ and ORIEN, the advantages of a single-site data warehouse have traditionally been speed of information flow (a consequence of fewer interoperability and regulatory barriers) and customizability (e.g., the ability to link with registry data, or create custom fields for NLP-derived data). However, national and international data-sharing platforms are becoming increasingly versatile and represent a superior solution for big data analyses going forward. This aligns with the National Cancer Institute Blue Ribbon Panel report calling for a National Cancer Data Ecosystem. The architecture of these broader initiatives may draw on the experience of single-site data repositories like our own.

In conclusion, clinical research data warehouses which draw upon multiple data sources and have the capacity to expand to derived data streams such as from NLP, present a promising platform for driving observational research. Oncology is a natural starting place for this approach, as there are already valuable aggregated datasets available through registries at the institutional and state level. The value is in combining breadth and depth—linking large-scale registry data to the granularity of EHR data in order to enable more powerful research questions to be answered. The experience of local data warehouses may help to inform the architecture of cross-institutional data-sharing initiatives.
